# Quality of Life in Young Adults after Flatfoot Surgery: A Case-Control Study

**DOI:** 10.3390/jcm10030451

**Published:** 2021-01-24

**Authors:** Nicolò Martinelli, Alberto Bianchi, Lorenzo Prandoni, Emanuele Maiorano, Valerio Sansone

**Affiliations:** 1 IRCCS Galeazzi, Via R. Galeazzi 4, 20100 Milan, Italy; bianchi_alberto@hotmail.com (A.B.); lorenzo.prandoni@gmail.com (L.P.); emanuele.maiorano@yahoo.it (E.M.); valerio.sansone@unimi.it (V.S.); 2 Department of Orthopedics, University of Milan, 20122 Milan, Italy

**Keywords:** flatfoot surgery, pain measurement, quality of life, health status

## Abstract

The true impact of surgery for flatfoot deformities on patient’s quality of life and health status remains poorly defined. The aim of this study is to evaluate the quality of life and the return to daily tasks and sports or physical activities in young adults after surgical correction of flatfoot deformity. Patients treated for bilateral symptomatic flat foot deformity were retrospectively studied. The healthy control group comprised a matched reference population with no history of foot surgery or trauma that was voluntary recruited from the hospital community. All subjects were asked to fill out questionnaires centered on the assessment of the health-related quality of life (Short-form 36; SF-36) and physical activity (International Physical Activity Questionnaire; IPAQ). Most study group SF-36 subscales were lower when compared to the control group. Among the study group, post-operatively, 36.6% of patients managed to resume low levels of sports activity, 40% were sufficiently active and were able to perform moderate sports activity (an activity that requires moderate physical effort and which forces the patient to breathe with a frequency only moderately higher than normal), while 23.3% of them were active or very active and were able to perform intense physical activity. Most IPAQ scores were statistically different from the control group. The present study suggests that patients treated with medializing calcaneal osteotomy and navicular-cuneiform arthrodesis for symptomatic flafoot had lower levels of quality of life and physical activity when compared to healthy subjects. After surgery, patients showed a significant improvement in the clinical scores.

## 1. Introduction

Flaftoot deformity is a complex disease characterized by the decrease of the medial longitudinal arch and the valgus deformity of the hindfoot. Although there is a paucity of data regarding the epidemiology of adult flatfoot, this condition seems to be very common among the world population [1]. It is estimated that it affects 5 million people in the United States, while in the UK, its prevalence among females over 40 years old is over 3% [2]. In general, the prevalence of flatfoot among adults is 3–10% [3].

Pediatric flatfoot deformities tend to spontaneously correct during the first decade of life and generally do not need specific treatment [4]. In adults, flat feet can allow for a normal quality of life or it can become progressively symptomatic, causing pain and disability. If left untreated, symptomatic deformities may negatively affect quality of life, autonomy, and personal well-being [5].

In the presence of a flexible symptomatic flatfoot, the first treatment attempt should be nonoperative with an orthotic device, nonsteroidal anti-inflammatory drug (NSAIDs), and physical therapy [6]. Patients who are unresponsive to conservative treatment may benifit from a surgical correction of the deformity.

Some reports have shown a positive effect of surgery on quality of life and the general health status in the context of surgical outcomes [7,8]. However, reports on this issue are normally centered on surgical aspects and lack healthy control subjects. Therefore, the true impact of surgery for flatfoot deformities on patient quality of life and health status remains poorly defined.

Patients undergoing an operative treatment may have good functional outcomes, but the improvement of the patient’s quality of life is the ultimate goal of every procedure and should be thoroughly verified as well. Our hypothesis is that surgical flatfoot correction could significantly ameliorate patient quality of life, allowing the successfeul return to daily and physical activities, including sports. The aim of this study is to verify the aforementioned hypothesis and to evaluate the general health status of young adults after flatfoot surgery in comparison to the general population.

## 2. Materials and Methods

In this retrospective comparative case control study, patients treated for bilateral symptomatic flat foot deformity between November 2011 and February 2015 were compared with a matched-control group of healthy subjects with no history of flatfoot deformity.

The study was conducted according to the Declaration of Helsinki concerning medical research, and written informed consent was obtained from all patients and healthy subjects of the control group. Data were anonymized and stored in a protected database accessible only to one researcher.

Patients in this study were recruited from a population of patients undergoing routine follow-up at the outpatient department. Patients were invited to participate in the study if they fulfilled all of the following criteria: patients aged between 16 and 29 years, closure of the ossification cores, history of symptomatic flatfoot deformity surgically treated with medializing calcaneal osteotomy and navicular-cuneiform arthrodesis, at least 12 months follow-up after reconstruction. Postoperative radiographs of both feet were assessed for detecting and exluding patients with complications (e.g., midtarsal non-union). Other exclusion criteria were: recurrence or residual flat foot deformity, history of severe foot pain in the previous six months.

A matched reference population with no history of foot surgery or trauma was voluntarily recruited from hospital community as the control group. Age, gender, and body mass index (BMI) were used as matching parameters.

### 2.1. Evaluation Scores

All patients and subjects were asked to fill out questionnaires centered on the assessment of health-related quality of life (HRQoL) and physical activity. For the study group, clinical data collection was performed during the pre-admission visit and after surgery at the last follow up visit. Participants were included after the surgery.

During the follow-up visit, HRQoL questionnaires were collected, and foot alignment was clinically assessed with the foot posture index (FPI). Normal alignments were considered for FPI values between 0 and +5 [9]. To evaluate the HRQoL, the SF-36 score was chosen as an appropriate measuring instrument. The SF-36 is a clinical score used to evaluate the patient’s general health status [10]. It is a generic, multi-dimensional questionnaire divided into 36 questions. The result obtained from the answers is represented by 8 scores/scales, each one representing a quantification of a specific aspect of the state of health, and by two indices that summarize the overall assessments of physical (PCS) and mental health (MCS). Higher scores indicate a better perceived health level. SF-36 data were collected during the follow-up examination at least 12 months postoperatively. To correlate the HRQoL with the clinical situation, the following scores were analyzed pre-and post-operatively at the last follow-up: the American Orthopedic Foot and Ankle (AOFAS) score and visual analog scale (VAS) to evaluate pain [11]. The International Physical Activity Questionnaire (IPAQ) and the University of California at Los Angeles (UCLA) activity score were used post-operatively to assess self-administrated physical activity during the preceding week [12,13]. For the IPAQ, each activity domain was weighed by its energy requirements defined in metabolic equivalent of tasks (METs) to yield a score in MET-min/week. The amount of activity was classified into three categories: low, moderate, or high physical activity level (IPAQ scoring protocol; IPAQ Consensus Group 2005) [14]. Subjects of the control group were clinically assessed with the FPI and filled out the SF-36, the IPAQ, and the UCLA questionnaires. Subjects with pronated or supinated feet were excluded from the study.

### 2.2. Statistical Analysis

All analyses were performed using SPSS (SPSS Statistics V20; SPSS, Inc, an IBM Company, Chicago, IL, USA). Starting from the analysis of literature [7,15], a priori power analysis for a two-tailed paired Student’s *t*-test (alfa  =  0.05, power  =  0.8, mean difference of 8.7  ±  5.5 for the physical functioning subscale of SF-36) indicated a minimum sample size of 26 subjects. After the assessment of the distribution pattern of the variables, a comparison of mean between the two groups was performed using a parametric test (independent *t*-test) for normally distributed variables, whereas their non-parametric counterparts (Mann–Whitney U test) were used for non-normally distributed variables. Correlations were computed between the HRQoL (SF-36) and the clinical result (AOFAS, VAS) for the study group based on the Spearman correlation coefficient. Statistical significance was set at 0.05 *p*-value.

## 3. Results

A sample of 30 patients and 31 matched reference subjects was available at the last follow-up and was included in this study. One patient of the study group, who initially accepted to participate, did not come at the follow up visit. The study group included 13 (43.3%) females and 17 (56.6%) males with the mean age of 21.9 ± 7.8 years. The control group included 15 (48.3%) females and 16 (51.7%) males with the mean age of 24.2 ± 2.8 years. In the study group, the mean FPI at the last follow-up was 2.6 ± 1.7. Age, gender, and BMI were similar in both groups (*p* > 0.05).

In the study group, the average AOFAS score significantly increased from 48.1 ± 22.2 (range 25 to 71) pre-operatively to 77 ± 17.2 (range from 58 to 95) post-operatively (*p* < 0.01). The VAS scale also shows a marked improvement: from 6.5 ± 2.1 (range 3 to 10) pre-operatively to 2.9 ± 1.9 (range from 0 to 7) post-operatively (*p* < 0.01). Before surgery, most patients were able to participate in modest physical activities, such as walking, limited housework, and limited shopping (UCLA average value 2.6); after surgery, patients were able to regularly participate in moderate physical activities (UCLA average value 6.1) (*p* < 0.01).

At the last follow-up, the operated patients show an average mental (MCS) and physical (PCS) component of SF-36 of 69.7 ± 5.8 and 74.5 ± 24.2, respectively. Most SF-36 subscales scores were significantly lower in the study group when compared to the control group with the exception of physical functioning (PF), social functioning (SF), and role-emotional (RE) (Table 1).

Significant correlations were found between clinical scores (i.e., AOFAS, VAS) and SF-36 domains (role-physical, bodily pain, general health, vitality, and mental health) (*p* <0.01).

After surgery, all patients were able to walk without difficulty and participate in sports activities. The UCLA score improved from 2.6  ±  1.9 (SD) preoperatively to 6.1  ±  1.7 (SD) postoperatively (*p* <  0.01). The final UCLA score, however, was significantly lower than the control group value (6.1  ±  1.7 vs. 7.5 ± 1.1; *p* < 0.01).

Among the study group, post-operatively, 36.6% of patients managed to resume low levels of sports activity, 40% were sufficiently active and were able to perform moderate sports activity (an activity that requires moderate physical effort and which forces the patient to breathe with a frequency only moderately higher than normal), while 23.3% of them were active or very active and were able to perform intense physical activity (an activity that requires high physical effort and which forces the patient to breathe at a much higher rate than normal).

In the study group, the average of the “total” domain was 1494.3 ± 1016.9 MET-min/week.

Patients who had a “low” physical activity level walked an average of 174.9 ± 70.8 Met-min/week and performed moderate intensity physical activity with an average of 175.0 ± 138.0 Met-min/week and physical activity at vigorous intensity with an average of 288.0 ± 297.5.

Patients who had a “moderate” physical activity level walked an average of 283.2 ± 206.4 Met-min/week and performed moderate intensity physical activity with an average of 389.0 ± 245.3 Met-min/week and physical activity at vigorous intensity with an average of 998.0 ± 458.6.

Patients who had a “high” physical activity level walked an average of 343.8 ± 164.4 Met-min/week and performed moderate intensity physical activity with an average of 444.2 ± 299.3 Met-min/week and physical activity at vigorous intensity with an average of 1829.4 ± 518.0.

Most IPAQ scores were statistically different from the control group (Table 2).

## 4. Discussion

The main objective of the study was to assess the quality of life and the return to physical activities in young adults operated for symptomatic flatfoot deformity with the same surgical technique.

Surgery for flatfoot deformity is usually indicated in the presence of symptoms affecting the activities of daily life and after the failure of prolonged conservative therapies. Among the adult population, bony procedures (e.g., osteotomies) with additional soft-tissue procedures are satisfactory approaches for the management of symptomatic flexible flatfoot deformities [16]. Navicular-cuneiform fusion is a common procedure that may be used to balance a symptomatic flatfoot, providing medial column stability and alignment, although reducing motion of the midtarsal joints [17,18]. In the literature, good clinical and radiological outcomes are reported for this technique with and without different bony procedures (e.g., medializing calcaneal osteotomy) [19]. The clinical improvement is confirmed throughout this study.

To verify the effectiveness of surgery in the post-operative period, the surgically treated group was compared to a healthy control group without foot deformities. It emerged that, while surgical treatment leaded to an improvement in the clinical outcome scores, patients’ perceived pain and quality of life were still impaired in comparison to the control group.

In a study conducted by Pita-Fernandez et al. on a random adult population sample, patients with flat foot showed lower quality of life and foot function than patients not suffering from the disorder; that effect was still present after adjusting for age, sex, and comorbidity using the Foot Health Status Questionnaire (FHSQ) and Foot Function Index (FFI) questionnaires [20]. In pediatric populations, patients with flexible flatfoot were found to have worse quality of life compared to children with normal feet as a result of the abnormalities in different kinematic parameters (e.g., reduced stride length and consequently walking speed) [21].

Conversely different results were found by other authors. López-López et al. carried out a cross-sectional study taimed to determine the relationships between relatively high, low, and normal ranges of feet arches and quality of life [5]. FHSQ results of three groups did not show any statistically significant difference (*p* > 0.05) for any domains of specific foot (pain, function, general health, and footwear) and general (general health, physical activity, social function, and vigor) health-related quality of life [5]. In a previous study, Esterman et al. in Australian recruits of area forces showed how foot alterations were not related to pain, injury, or functionality, although flatfoot was associated with a lower subjective feeling of physical health than those with normal foot [22]. In another study, Bandlissi et al. did not find any association between foot alteration, pain, and functionality [23].

In this study, we found lower quality of life levels for most SF-36 parameters in the operated patients compared to the healthy control group except for the subscales concerning physical functioning (PF), social functioning (SF), and role-emotional (RE). Similar results were observed for data concerning physical activity. All operated patients reported lower levels of physical activity compared to the control group. In a previous study, Martinelli et al. reported the return to sport rate after subtalar arthroreisis for pediatric symptomatic flatfoot deformity [24]. Forty-five patients returned to sports after surgery with no difference in duration, type of sports activities, or frequency, but the emotional status as well as the participation in physical activities and footwear issues improved. Nevertheless, the lack of a control group prevented the determination of the subjects’ physical activity level.

Psychological aspects could affect the sport activity levels in a symptomatic flatfoot patient who underwent surgically interventions. A psychological problem has largely been advocated as the reason for a delayed or absent return to sport among athletes after anterior cruciate ligament reconstruction. In this group of patients, fears of reinjury and reinjury anxiety are common psychological constructs that may not be related to pain. They could instead be related to the potential consequences of injury (e.g., additional surgery and more time in rehabilitation) [25]. Similar worries could be present among symptomatic flatfoot patients after surgery, leading them to avoid activities they perceive as dangerous to their new, post-surgical physical balance.

Several authors have argued the importance of using specific instruments for measuring foot disorders, as general health measures struggle to isolate specific orthopedic outcomes via changes in other health conditions that may be unrelated [26]. However, the use of generic questionnaires for studying the quality of life and foot function is extensively documented in the literature [27]. Therefore, we decided to measure the quality of life using a general health score (i.e., SF-36) together with a foot pain/function with site specific scores (i.e., AOFAS score). In this study, most of the SF-36 subscales showed significant correlations with foot and ankle specific outcome measure (i.e., AOFAS score). These findings partially overlapped with the results from a previous study. Ceccarelli et al. showed moderate correlation between SF-36 “Bodily Pain” and “Physical Function” subscales with the AOFAS score results [28]. Similar findings were reported in the study of Westphal et al. when determining correlations between the AOFAS score and the SF-36 subscale in patients with a sequelae of intraarticular calcaneal fractures [29]. In patients affected by chronic conditions of foot and ankle requiring operative intervention, SooHoo et al. showed satisfactory levels of responsiveness of SF-36 subscales (i.e., bodily pain sub-scale and physical component summary scale) approaching that of the AOFAS clinical rating system [27].

Surgery for symptomatic flatfoot deformity has been proven to provide pain relief and to improve patient quality of life and physical activity. Outcomes of flatfoot reconstruction in the young adult patient have been previously reported in a study of Day et al.; the authors reported an improvement in physical activity and quality of life measured with the Patient-Reported Outcomes Measurement Information Systems in a cohort of 19 patients with mean age 20.8 years [30]. In a recent study, Tejero et al. reported an improvement in the quality of life measured with SF-36 questionnaire in a cohort of sixty-two patients (67 feet) for symptomatic stage III flatfoot deformity who were treated using a modified double arthrodesis by a minimally invasive technique [7]. Nevertheless, no comparison with a healthy control group was performed.

The present study has some limitations. First, the SF-36 and the IPAQ surveys for assessing participants‘ quality of life and total physical activity were administered only at the last follow-up, which may be a relevant factor in assessing surgery effectiveness in symptomatic flatfoot deformity. However, clinical outcome scores (i.e., AOFAS score and VAS for pain) showed a significant improvement after surgery. Second, weightbearing x-rays were not collected at the last follow-up because of bone consolidation observed post-operatively. Clinically, patients had a satisfactory alignment assessed with the FPI.

## 5. Conclusions

The present study suggests that patients treated with medializing calcaneal osteotomy and navicular-cuneiform arthrodesis for symptomatic flafoot had lower levels of quality of life and physical activity when compared to healthy subjects. After surgery, patients showed a significant improvement in the clinical scores.

## Figures and Tables

**Table 1:** Short-form 36 (SF-36) subscales in the two groups. In bold are the statistical significant differences. PF: physical functioning; RP: role-physical; BP: bodily pain; GH: general health; VT: vitality; SF: social functioning; RE: role-emotional; MH: mental health.

SF-36 Subscale	Study Group	Control Group	*p*-Value
PF	80.0 ± 21.0	87.2 ± 9.1	*p* > 0.05
RP	70.2 ± 33.3	87.4 ± 9.1	***p* < 0.01**
BP	73.3 ± 18.8	89.1 ± 6.0	***p* < 0.01**
GH	76.3 ± 9.9	93.4 ± 7.7	***p* < 0.01**
VT	79.6 ± 7.4	90.3 ± 9.4	***p* < 0.01**
SF	92.8 ± 9.3	88.2 ± 9.7	*p* > 0.05
RE	84.7 ± 31.6	88.8 ± 10.0	*p* > 0.05
MH	89.5 ± 9.5	96.5 ± 6.4	***p* < 0.01**

**Table 2:** Physical activity in the two groups. MET: metabolic equivalent of task. IPAQ: International Physical Activity Questionnaire.

IPAQ	Study Group	Control Group	*p*-Value
Total (MET-min/week)	1494.3 ± 1016.9	2048.3 ± 1038.3	0.03
Walking (MET-min/week)	186.2 ± 126.0	344.3 ± 173.1	<0.01
Moderate (MET-min/week)	255.3 ± 263.2	414.1 ± 234.3	<0.01
Vigorous (MET-min/week)	809.3 ± 577.6	1232.2 ± 850.5	0.04
Sitting time (hours/day)	3.3 ± 0.6	3.7 ± 0.8	0.05

## Data Availability

The datasets generated and analysed during the current study are not publicly available due to privacy reasons but are available from the corresponding author on reasonable request.
